# Incidence and Risk Factors of Lumbosacral Complications Following Long‐Segment Spinal Fusion in Adult Degenerative Scoliosis

**DOI:** 10.1111/os.14275

**Published:** 2024-10-28

**Authors:** Tinghua Jiang, Xinuo Zhang, Qingjun Su, Xianglong Meng, Aixing Pan, Hanwen Zhang, Yong Hai

**Affiliations:** ^1^ Department of Orthopedic Surgery, Beijing Chao‐Yang Hospital Capital Medical University Beijing China; ^2^ Department of Orthopedic Surgery Beijing Huairou Hospital Beijing China

**Keywords:** adult degenerative scoliosis, long floating fusion, lumbosacral complications, risk factors

## Abstract

**Purpose:**

Long‐segment spinal fusions are associated with lumbosacral complications (LSC), but the associated risk factors are not known. This study aimed to identify the risk factors for LSC after long‐segment instrumented fusion with distal fixation to the L5 vertebral body in adult degenerative scoliosis (ADS).

**Methods:**

We retrospectively evaluated 294 patients with ADS who underwent long‐segment floating fusion between January 2014 and March 2022, with follow‐up for at least 2 years. Patients were matched to the baseline data using fusion level > 5 as a grouping variable. Patients who completed matching were divided into two groups according to the presence or absence of LSC. Univariate logistic regression was applied to identify potential risk factors for LSC, and multivariate logistic regression was used to identify independent risk factors for postoperative LSC.

**Results:**

The overall incidence of LSC was 21.77% in the 294 patients, with disc degeneration in 28 (9.52%) and radiographic ASD in 44 (14.97%) patients. The mean time to LSC development after surgery was 26.91 ± 8.43 months. A total of 54 pairs of patients were matched and grouped, and the complication group had higher Oswestry Disability Index (ODI) and visual analog scale (VAS) scores at the last follow‐up. Multivariate analysis showed that gender (OR = 0.274, *p* = 0.026 [0.087, 0.859]); levels of fusion > 5 (OR = 3.127, *p* = 0.029 [1.120, 8.730]), main curve correction rate (OR = 0.009, *p* = 0.005 [0.000, 0.330]), and postoperative pelvic incidence minus lumbar lordosis (PI‐LL) > 15° (OR = 3.346, *p* = 0.022 [1.195, 9.373]) were independent risk factors for postoperative LSC. The area under the curve value of the prediction model was 0.804, with a 95% confidence interval of 0.715–0.892, indicating that the model had a high prediction accuracy. Collinearity statistics showed no collinearity between variables.

**Conclusion:**

Sex, level of fusion > 5, main curve correction rate, and postoperative PI‐LL > 15° were independent risk factors for the development of LSC after long‐segment floating fusion. These results will improve our ability to predict personal risk conditions and provide better medical optimisation for surgery.

## Graphical abstract

We retrospectively evaluated 294 patients with ADS who underwent long‐segment floating fusion between January 2014 and March 2022, with follow‐up for at least two years. Patients were matched to the baseline data using fusion level > 5 as a grouping variable. Patients who completed matching were divided into two groups according to the presence or absence of LSC. Univariate logistic regression was applied to identify potential risk factors for LSC, and multivariate logistic regression was used to identify independent risk factors for postoperative LSC.

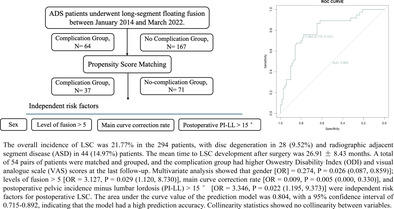

## 1 Introduction

Adult degenerative scoliosis (ADS) is a form of adult scoliosis defined by a scoliotic Cobb angle greater than 10°, with no history of adolescent idiopathic scoliosis in childhood or adolescence [1]. Although the estimated prevalence of ADS in the general population ranges from 30% to 60% [2], it may be as high as 64%–68% in the elderly [3]. Conservative treatment is usually the treatment of choice; however, long‐segment spinal fusion orthopedic surgery can be performed in patients with ADS who do not respond well to conservative treatments [4].

Surgical procedures correct coronal and sagittal imbalances by decompressing and maintaining spinal stability [5]. Although interbody fusion and fixation are recommended to improve spinal alignment in ADS, these procedures sacrifice spinal mobility. Recent studies have recommended avoiding long‐segment instrumentation in patients without substantial spinal imbalance [6]. As such, it remains controversial whether to preserve the connection between lumbar 5 and sacral 1 (L5‐S1) without preexisting lesions when performing fusion surgery in ADS patients. Spinal fusion ending in the fifth lumbar vertebra (L5) is also known as floating fusion. L5 was chosen as the lowest instrumented vertebra, preserving the mobility of L5‐S1 with the advantage of minimizing the fusion level and decreasing the pseudarthrosis formation rate [7].

Patients undergoing floating fusion are also at risk of complications, such as subsequent L5‐S1 disc degeneration and sagittal malalignment, with relevant studies reporting complication rates ranging from 15.91% to 69% [8]. No studies have previously separately discussed the risk factors for subsequent lumbosacral complications (LSC) after floating fusion in patients with ADS. As such, it is necessary to evaluate the risk factors for LSC in floating fusions in patients with ADS. Therefore, in this study, propensity matching was used to eliminate confounding factors, and logistic regression was used to find risk factors. This study aimed to retrospectively evaluate patient‐ and surgery‐related risk factors for subsequent LSC after long‐segment spinal fusion to L5 in patients with ADS.

## 2 Methods

### 2.1 Patients

This retrospective study enrolled 294 patients with ADS who underwent long‐segment floating fusion at a single centre between January 2014 and March 2022. This study was performed in accordance with the principles of the Declaration of Helsinki and was approved by the Institutional Review Board of Beijing Chao‐Yang Hospital (ID 2024‐Ke‐350).

The inclusion criteria were as follows: (1) Cobb angle > 10°, (2) posterior procedure only, (3) age greater than 45 years at the time of surgery, (4) surgery segments with pedicle screw instrumentation ≥ 3, (5) L5 as the lower instrumented vertebra (LIV); (6) Pfirrmann classification of L5‐S1 disc preoperative was 1 or 2, and (7) a minimum of 2 years of follow‐up.

The exclusion criteria were as follows: (1) history of previous lumbar surgery, (2) spinal tumors or infections, (3) adolescent idiopathic scoliosis, (4) ankylosing spondylitis, (5) Pfirrmann classification of the L5‐S1 disc before surgery as Grade 3, 4, or 5, and (6) incomplete imaging information and functional evaluation results.

### 2.2 Data Collection

Baseline data, including age, sex, body mass index (BMI), presence of osteoporosis, follow‐up time, smoking status, presence of diabetes, Pfirrmann classification of the L5‐S1 disc, and fusion level, were collected.

Radiographic data were collected before and after the surgery and at each subsequent follow‐up visit. Patients were asked to attend the clinic by telephone for follow‐up at 1, 3, and 6 months, and yearly after surgery. The radiographic data included in the statistical analysis were obtained at the final follow‐up. Anteroposterior and lateral standing whole‐spine radiographs (Philips Digital Diagnost, Zhejiang Province, China) were performed to evaluate ADS patients. All images were downloaded from the Picture Archiving and Communication System (PACS) and analyzed using dedicated software (Surgimap, Nemaris, Inc., New York, USA). The measured parameters included the Coob angle correction rate of the main curve, sagittal imbalance, sacral slope, pelvic incidence minus lumbar lordosis (PI‐LL), and L5‐S1 range of motion (ROM). All radiographic measurements were performed independently by two spine surgeons to reduce intra‐observer variability. Sagittal imbalance was defined as the horizontal distance from the C7 plumb to the posterosuperior corner of the sacrum; > 5 cm was regarded as sagittal imbalance [9]. Clinical outcomes, including the Oswestry Disability Index (ODI) and visual analog scale (VAS) scores, were collected preoperatively and at the final follow‐up.

### 2.3 Complications

The patients were divided into the complication and no‐complication groups according to the occurrence of LSC. In this study, LSC was defined as the presence of radiographic adjacent segment degeneration (ASD) or disc degeneration. Radiographic ASD was defined as > 3 mm loss of disc height, > 5° increase in posterior angulation, or > 3 mm progression of spondylolisthesis between previously reported preoperative and postoperative standing radiographs [10]. Patients meeting at least one of these criteria were diagnosed with radiographic ASD. Magnetic resonance imaging (MRI) was performed to evaluate disc degeneration before and after surgery at the L5‐S1 level according to the Pfirrmann classification [11]. A Pfirrmann classification of 3, 4, or 5 at follow‐up was defined as the presence of disc degeneration.

### 2.4 Statistical Analysis

Data analysis was performed using SPSS Statistics (version 23.0; IBM, Armonk, New, USA) and R software (version 4.1.2). Continuous variables are presented as the means and standard deviations, and categorical variables are presented as frequencies. Comparisons between quantitative data were performed using the *t*‐test, and comparisons between categorical data were performed using the chi‐square test. To eliminate the influence of follow‐up time on complications, baseline data from respondents who were followed up for less than 5 fusion levels and > 5 fusion levels were matched 1∶1 without replacement using the closest matching method with a caliper value set at 0.02. Univariate analysis was applied to identify the potential risk factors for LSC. Risk factors with a *p*‐value < 0.2 in univariate analysis were included in the multivariate analysis. Multivariate Logistic regression was used to identify the independent risk factors for LSC. Statistical significance was set at *p* < 0.05.

## 3 Results

### 3.1 Demographic and Procedural Data

A total of 294 patients with ADS who met these criteria were enrolled. Of the 294 patients, 64 (21.77%) presented with LSC at follow‐up, 28 (9.52%) patients presented with disc degeneration of Pfirrmann classification grades III, IV, or V, and 44 (14.97%) patients presented with radiographic ASD. The mean time to LSC development after surgery was 26.91 ± 8.43 months, and none of the patients required reoperation for LSC. To eliminate any differences, the baseline variables were matched according to whether the surgically fused level was greater than 5. A total of 108 patients completed the matching. The baseline parameters before and after propensity score matching (PSM) are listed in Table 1.

**TABLE 1:** Comparison of the baseline characteristics before and after PSM between the complication and no‐complication groups.

Characteristic	Before matching				After matching			
	Complication group	No complication group	*χ* ^2^	*p*	Complication group	No‐complication group	χ^2^	*p*
	*N* = 64	*N* = 167			*N* = 37	*N* = 71		
Age (years)			5.987	0.14			2.256	0.133
< 65	44 (51.76%)	140 (66.99%)			19 (51.35%)	47 (66.20%)		
≥ 65	41 (48.24%)	69 (30.01%)			18 (48.65%)	24 (33.80%)		
Gender			20.621	< 0.001			9.656	0.002
Male	22 (25.88%)	115 (55.02%)			6 (16.22%)	33 (46.48%)		
Female	63 (74.12%)	94 (44.98%)			31 (83.78%)	38 (53.52%)		
BMI (kg/m^2^)			135.831	< 0.001			1.241	0.265
< 25	73 (85.88%)	30 (14.35%)			25 (67.57%)	55 (77.46%)		
≥ 25	12 (14.12%)	179 (85.65%)			12 (32.43%)	16 (22.54%)		
Osteoporosis			27.597	< 0.001			11.809	< 0.001
Yes	59 (69.41%)	194 (92.82%)			11 (29.73%)	4 (5.63%)		
No	26 (30.59%)	15 (7.18%)			26 (70.27%)	67 (94.37%)		
Follow‐up (months)			5.213	0.22			0.666	0.414
< 36	41 (48.24%)	71 (33.97%)			21 (56.76%)	46 (64.79%)		
≥ 36	44 (51.76%)	138 (66.03%)			16 (43.24%)	25 (35.21%)		
Smoking			0.001	0.981			1.241	0.265
Yes	53 (62.35%)	130 (62.20%)			16 (43.24%)	23 (32.39%)		
No	32 (37.65%)	79 (37.80%)			21 (56.76%)	48 (67.61%)		
Diabetes			4.580	0.032			1.138	0.286
Yes	51 (60.00%)	152 (72.73%)			13 (35.14%)	18 (25.35%)		
No	34 (40.00%)	57 (27.27%)			24 (64.86%)	53 (74.65%)		
Pre‐operative Pfirrmann's grade							0.001	0.976
I					22 (59.46%)	42 (59.15%)		
II					15 (40.54%)	29 (40.85%)		

### 3.2 Radiographic and Clinical Outcomes

The correction rate was 73.77 ± 13.54% in the complication group and 79.01 ± 11.09% in the no‐complication group, and the difference between the two groups was statistically significant (*p* = 0.033). Nineteen patients (51.35%) in the complication group had postoperative PI‐LL > 15°, it was more than 14 patients (19.72%) in the no‐complication group, and the difference between the two groups was statistically significant (*p* < 0.001). The remaining parameters were not significantly different between the two groups (Table 2).

**TABLE 2:** Radiographic and follow‐up parameters (after PSM).

Characteristic	Complication group	No‐complication group	*χ* ^2^ */T*	*p*
	*N* = 37	*N* = 71		
Levels of fusion > 5 (%)	22 (59.46)	32 (45.07%)	2.014	0.156
Main curve correction rate (%)	73.77 ± 13.54	79.01 ± 11.09	2.157	0.033
Preoperative PI‐LL > 15°	19 (51.35%)	40 (56.34%)	0.244	0.621
Postoperative PI‐LL > 15°	19 (51.35%)	14 (19.72%)	11.471	< 0.001
Preoperative sacral slope (°)	35.32 ± 9.47	36.46 ± 7.92	0.663	0.509
Postoperative sacral slope (°)	34.92 ± 8.56	35.37 ± 6.73	0.298	0.766
Preoperative L5‐S1 ROM (°)	4.88 ± 1.10	4.68 ± 1.32	−0.786	0.433
Postoperative L5‐S1 ROM (°)	5.27 ± 1.90	4.75 ± 1.35	−1.483	0.144
Preoperative sagittal imbalance (*n* %)	12 (32.43%)	29 (40.85%)	0.731	0.393
Postoperative sagittal imbalance (*n* %)	9 (24.32%)	13 (18.31%)	0.542	0.461
Preoperative VAS	6.92 ± 1.19	6.75 ± 1.33	−0.663	0.254
VAS at last follow‐up	3.11 ± 1.31	2.66 ± 0.92	−2.056	0.021
Preoperative ODI	62.43 ± 2.50	62.77 ± 2.73	0.636	0.263
ODI at last follow‐up	28.22 ± 6.12	25.48 ± 8.25	−1.949	0.027

*Note*: Values are expressed as the mean ± standard deviation or number.

Abbreviations: BMI, body mass index; L5‐S1, the segment from fifth lumbar vertebra to first sacral vertebra; ODI, Oswestry disability index; VAS, visual analogue scale.

Preoperative VAS was 6.92 ± 1.19 in the complication group and 6.75 ± 1.33 in the no‐complication group, and there was no significant difference between the two groups. Preoperative ODI was 62.43 ± 2.50 in the complication group and 62.77 ± 2.73 in the no‐complication group, with no significant difference between the two groups. VAS at last follow‐up was 3.11 ± 1.31 in the complication group and 2.66 ± 0.92 in the No Complication Group, and the difference between the two groups was statistically significant (*p* = 0.021). ODI at last follow‐up was 28.22 ± 6.12 in the complication group and 25.48 ± 8.25 in the no‐complication group, with no significant difference between the two groups (*p* = 0.027) (Tables 2 and 3).

**TABLE 3:** Results of the univariate analysis.

Variables	*B*	SE	Wald	df	*p*	OR	95% CI for EXP(*B*)	
							Lower	Upper
Age	0.618	0.414	2.232	1	0.135	1.855	0.825	4.174
Gender	−1.501	0.506	8.818	1	0.003	0.223	0.083	0.600
BMI	−0.501	0.452	1.229	1	0.268	1.605	0.681	3.999
Osteoporosis	1.958	0.628	9.725	1	0.002	7.087	2.070	24.262
Smoking	0.464	0.418	1.233	1	0.267	1.590	0.701	3.605
Diabetes	0.467	0.439	1.129	1	0.288	1.595	0.674	3.773
Follow‐up	0.338	0.415	0.664	1	0.415	1.402	0.622	3.159
Levels of fusion > 5	0.581	0.411	1.996	1	0.158	1.787	0.799	4.001
Main curve correction rate	−3.508	1.699	4.263	1	0.039	0.030	0.001	0.837
Preoperative PI‐LL > 15°	−0.201	0.407	0.244	1	0.621	0.818	0.369	1.816
Postoperative PI‐LL > 15°	1.458	0.444	10.783	1	0.001	4.298	1.800	10.261
Preoperative sacral slope	−0.016	0.024	0.445	1	0.505	0.984	0.939	1.032
Postoperative sacral slope	−0.008	0.028	0.090	1	0.764	0.992	0.939	1.047
Preoperative L5‐S1 ROM	0.129	0.163	0.624	1	0.430	1.138	0.826	1.567
Postoperative L5‐S1 ROM	0.211	0.130	2.610	1	0.106	1.235	0.956	1.594
Preoperative sagittal imbalance	−0.364	0.426	0.728	1	0.394	0.985	0.302	1.603
Postoperative sagittal imbalance	0.361	0.491	0.539	1	0.463	1.434	0.548	3.753

*Note*: A *p* value of 0.05 represents the threshold for statistically significance.

Abbreviations: BMI, body mass index; CI, confidence interval; L5‐S1, the segment from fifth lumbar vertebra to first sacral vertebra; OR, odds ratio; PI‐LL, pelvic incidence minus lumbar lordosis; ROM, range of motion; SE, standard error.

### 3.3 Univariate and Multivariate Logistic Regression Analysis

**FIGURE 1:**
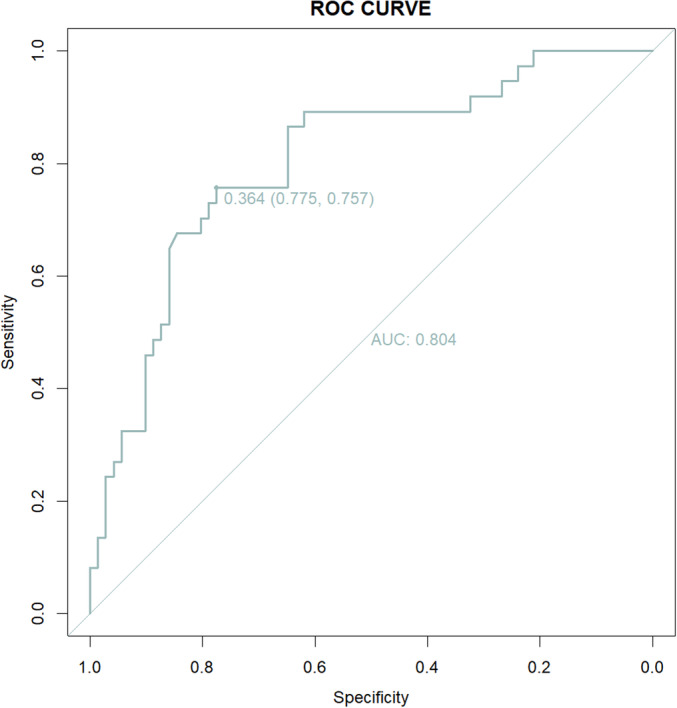
Receiver operating characteristic (ROC) curve of the predictive model.

Univariate logistic regression analysis showed that age > 65, gender, osteoporosis, levels of fusion > 5, correction rate, postoperative P I‐LL > 15°, and postoperative L5‐S1 ROM were potential risk factors for LSC after surgery (Table 4). Multivariate logistic regression analysis was used to analyze the above potential risk factors to determine the independent risk factors for LSC. Independent risk factors for developing LSC included sex (OR = 0.274, *p* = 0.026 [0.087, 0.859]), levels of fusion > 5 (OR = 3.127, *p* = 0.029 [1.120, 8.730]), main curve correction rate (OR = 0.009, *p* = 0.005 [0.000, 0.330]), and postoperative PI‐LL > 15° (OR = 3.346, *p* = 0.022 [1.195, 9.373]) (Table 4). The AUC value was 0.804 with a 95% confidence interval of 0.715–0.892, indicating a high predictive accuracy of this model (Figure 1). Collinearity statistics showed Variance inflation factor (VIF) < 5 and Tolerance > 0.1 for all variables. It can be considered that there is no collinearity problem between variables (Table 5).

**TABLE 4:** Results of the multivariate analysis.

Variables	*B*	SE	Wald	df	*p*	OR	95% CI for EXP(*B*)	
							Lower	Upper
Age > 65	0.262	0.500	0.275	1	0.600	1.300	0.487	3.467
Gender	−1.294	0.583	4.935	1	0.026	0.274	0.087	0.859
Osteoporosis	1.225	0.738	2.753	1	0.097	3.403	0.801	14.46
Levels of fusion > 5	1.140	0.524	4.739	1	0.029	3.127	1.120	8.730
Main curve correction rate	−5.332	2.155	6.120	1	0.013	0.005	0.000	0.330
Postoperative PI‐LL > 15°	1.208	0.526	5.282	1	0.022	3.346	1.195	9.373
Postoperative L5‐S1 ROM	0.215	0.159	1.837	1	0.175	1.240	0.908	1.693

*Note*: A *p* value of 0.05 represents the threshold for statistically significance.

Abbreviations: CI, confidence interval; L5‐S1, the segment from fifth lumbar vertebra to first sacral vertebra; OR, odds ratio; PI‐LL, pelvic incidence minus lumbar lordosis; SE, standard error.

**TABLE 5:** Collinearity statistics.

Variables	Collinearity statistics	
	Tolerance	VIF
Gender	0.990	1.010
Levels of fusion > 5	0.966	1.035
Main curve correction rate	0.963	1.039
Postoperative PI‐LL > 15°	0.954	1.048

Abbreviations: PI‐LL, pelvic incidence minus lumbar lordosis; VIF, Variance inflation factor.

## 4 Discussion

This study aimed to identify risk factors associated with LSC following long‐segment floating fusion in patients with ADS. In this study, 64 of 294 patients (21.77%) who underwent ADS correction surgery (long‐segment floating fusion) presented with LSC. Of these, 28 (9.52%) had disc degeneration of Pfirrmann classification grades III, IV, or V, and 44 (14.97%) had radiographic ASD. After matching patients' baseline data, sex, levels of fusion > 5, correction rate, and postoperative PI‐LL > 15° were independent risk factors for LSC after floating fusion in the entire patient population by multivariate logistic regression analysis.

### 4.1 Fusion Levels > 5

In the present study, fusion levels > 5 were found to be an independent risk factor for postoperative LSC. Fusion makes the spine whole, forming a structure resembling a large lever arm [12]. The longer the fusion level, the greater is the cantilever force of the large‐arm lever on the fusion bottom [13], which ultimately leads to high stress at the bottom of the construct and increases the risk of LSC. Spine surgeons must choose long‐segment lumbar fusion as patients with ADS are often older and have more rigid scoliotic curves that are difficult to correct. However, this is contradicted by the higher shear stress associated with long‐segment fusion and the increased risk of disc damage and degradation at the L5‐S1 junction after long‐segment fusion. Surgical management of ADS is challenging for spine surgeons because of the high incidence of postoperative L5‐S1 degeneration and revision rates [7]. For ADS patients, preoperative planning and L5‐S1 assessment are necessary. Risky long‐segment fusion may have the opposite effect. This study innovatively identified LSC risk factors after long‐segment floating fusion. By avoiding these factors, long‐segment floating fusion can be made safer and more effective.

### 4.2 High Main‐Curve Correction Rate

A high main‐curve correction rate was a protective factor against LSC following floating fusion although the OR for this factor was low (OR = 0.009, *p* = 0.021; OR = 0.000, *p* = 2.021). The curvature of the spine in patients with ADS can lead to a shift in the centre of gravity [14]. When the centre of gravity of the spine shifts, the lumbosacral region, which acts as the terminal fixation space, bears greater pressure that the spine conducts downward. Increased disc pressure leads to increased disc deformation [8]. These factors increase the incidence of disc degeneration and radiographic ASD, which in turn lead to the development of LSC. Therefore, we recommend that the correction rate of the main curve be corrected by osteotomy and other means to reduce the incidence of LSC in patients with ADS using floating fusion as much as possible.

### 4.3 Sex Factor

Although ADS is a degenerative spinal disease in all adults, prior studies have shown that female patients are predominant, accounting for twice as many of the population as male patients [14, 15]. Previous studies have further reported a trend toward an increased incidence of ADS in female populations [15]. Logistic regression analysis showed that male sex was a protective factor among the independent risk factors associated with the degeneration of adjacent segments of the lumbosacral region after floating fusion (OR = 0.291, *p* = 0.027 [0.097, 0.872]). Previous studies have also shown that the LL is significantly higher in women than in men [16]. Women experience the highest spinal load relative to men at work and during activities [17]. These factors lead to women having higher lumbosacral shear forces, which, in turn, leads to an increased probability of lumbosacral degeneration. In addition, women had higher fatty infiltration and lower paravertebral muscle cross‐sectional areas than men. The lack of muscle protection further accelerates lumbosacral degeneration [18], and female patients are more likely to develop LSC after surgery.

### 4.4 Postoperative PI‐LL > 15°

The PI‐LL is an important spinal sagittal parameter. The results of the present study showed that postoperative PI‐LL > 15° was an independent risk factor for LSC in ADS patients undergoing long‐floating fusion. Recovery of PI‐LL mismatch has previously been shown to be strongly associated with better prognosis in patients with spinal deformity [19], Severe PI‐LL mismatch is strongly associated with postoperative low back pain, leg pain, and leg numbness [20]. Patients who develop adjacent segment degeneration usually have higher PI and lower LL, and the chance of adjacent segment degeneration increases greatly if fusion surgery is performed without correcting the PI‐LL mismatch [21]. As such, for patients with ADS undergoing long‐floating fusion, detailed preoperative planning should be performed, and the potential PI‐LL mismatch should be corrected as much as possible during surgery. If correction of the PI‐LL mismatch cannot be achieved, L5‐S1 fusion should be chosen.

### 4.5 Strengths and Limitations

The strengths of this study are the following aspects. The large sample size included in this study provides a robust dataset for statistical analysis. Follow‐up of no less than 2 years ensured long‐term evaluation of postoperative outcomes. Finally, propensity score matching was employed in this study to minimize confounding variables and enhance the reliability of the results.

This study had several limitations. First, this was a single‐centre retrospective study, which may have reduced the generalisability of the results. Second, other factors, such as patient comorbidities, changes in spinopelvic alignment, and patient clinical outcomes, should also be subcategorised and investigated. Finally, this study was retrospective and could not further investigate the correlation between the time of LSC appearance and imaging parameters. Large multi‐institutional prospective studies are required to validate these findings.

## 5 Conclusion

Level of fusion > 5 and postoperative PI‐LL > 15° were identified as independent risk factors for the development of LSC after long‐floating fusion in ADS patients, and male gender and a high rate of main curve correction are independent protective factors for LCS. We recommend careful preoperative identification of patients with long‐segment floating fusions, correction of the PI‐LL mismatch at the time of surgery whenever possible, and improvement in the rate of major curve correction. Lumbosacral fusion was used whenever possible in patients in whom the PI‐LL mismatch could not be corrected or who were at a high risk of LSC.

## Author Contributions

Writing – original draft: Tinghua Jiang and Xinuo Zhang. Supervision: Yong Hai. Formal analysis: Qingjun Su. Data curation: Xianglong Meng. Writing – review and editing: Aixing Pan and Hanwen Zhang. The authors read and approved the final manuscript.

## Conflicts of Interest

The authors declare no conflicts of interest.
